# Supplementary material for: Gut microbiota-derived butyrate primes systemic immunity in honey bees by mediating lipid metabolic reprogramming
Source: Nat Commun. 2026 Feb 2;17:2924. doi: 10.1038/s41467-026-69073-0 (PMC13031938; doi:10.1038/s41467-026-69073-0)
Supplement: Supplementary file 1 — Supplementary Information [file 41467_2026_69073_MOESM1_ESM.pdf]

Gut microbiota-derived butyrate primes systemic immunity in honey bees by mediating lipid metabolic reprogramming

Supplemental information

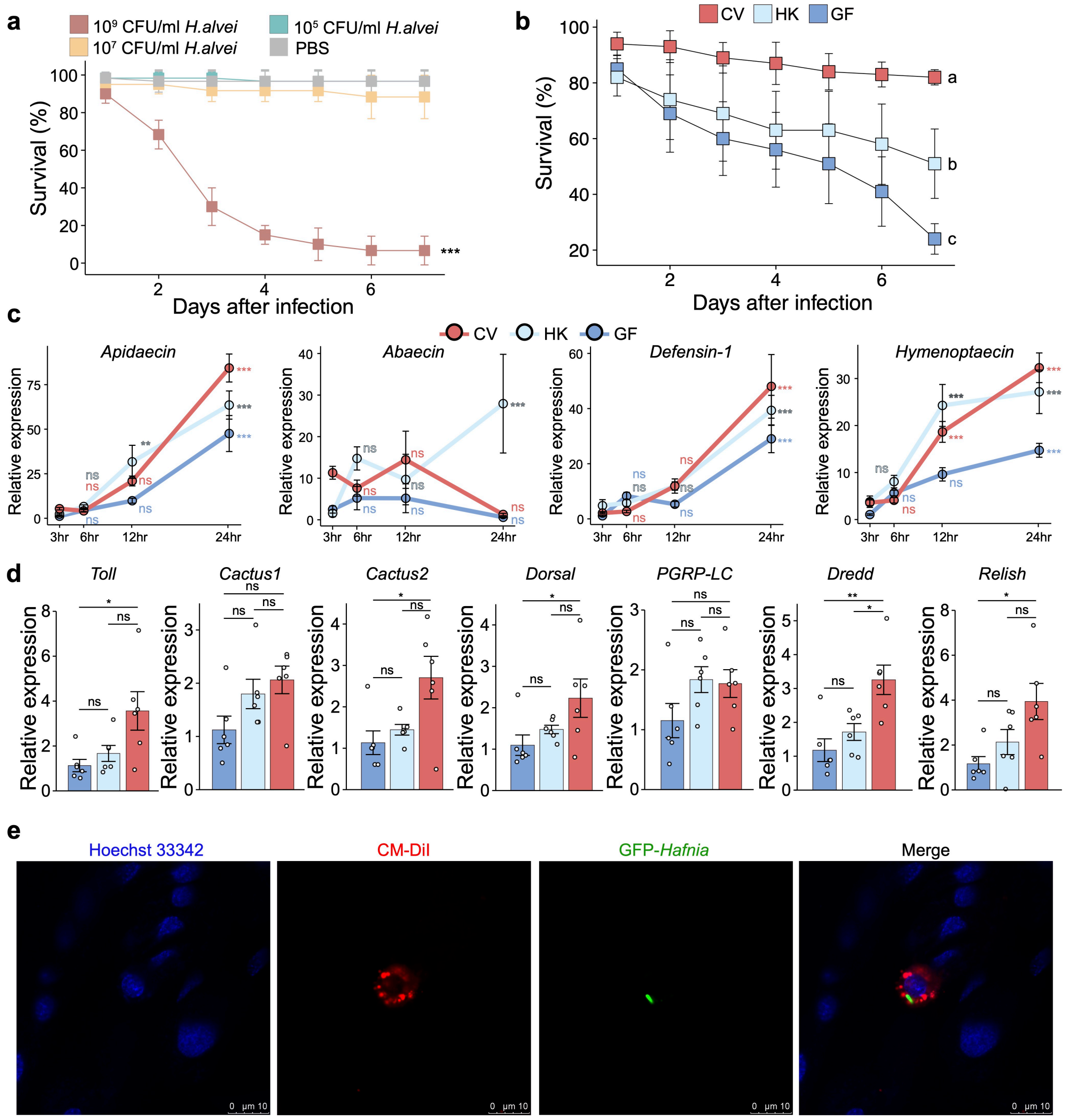

Supplementary Fig. 1 | Survival rate after *Hafnia* infection and the effect of gut microbiota on immune response. a A notable decline in survival rates among bees exposed to *Hafnia* at 10^9 CFU/ml. PBS was used as a control treatment. Bars represent mean ± s.d. n = 3 replicates for each group, with 20 individuals for each replicate. Two-sided Mantel-Cox test. ****P* < 0.001. **b** Survival rates for GF, HK, and CV bees post *Hafnia* injection. These are independent replicates of Fig. 1b. Bars represent mean ± s.d. from five biological replicates (20 individuals per replicate).

Two-sided Mantel-Cox test. Different letters above bars indicate statistically significant differences among treatments ($P < 0.05$). **c** Temporal dynamics of AMP gene expression following *Hafnia* injection. Relative expression levels of *Apidaecin*, *Abaecin*, *Defensin-1*, and *Hymenoptaecin* were measured in the fat body of GF, HK, and CV honey bees at 3 hr, 6 hr, 12 hr, and 24 hr post infection. Expression values were normalized to GF at 3 hr to illustrate time-course changes. Data are shown as mean ± s.e.m. n = 6 individuals per group from 3 cup cages. Two-way ANOVA with the Tukey's multiple comparisons test. ns: not significant, *$P < 0.05$, **$P < 0.01$, ***$P < 0.001$. **d** The expression of genes in IMD and Toll pathways in the fat bodies of GF, HK, and CV bees 3 hr post-infection. Gene expression was quantified using qPCR as shown in Fig. 1. Bars represent mean ± s.e.m. n = 6 individuals per group from 3 cup cages. One-way ANOVA with the Tukey post hoc test or Kruskal–Wallis test with Dunn's post hoc test, depending on data normality. *$P < 0.05$, **$P < 0.01$, ***$P < 0.001$. **e** Confocal microscopy images of bee hemocytes stained with CM-DiI (red) and Hoechst 33342 (blue; nuclear stain), showing GFP-*Hafnia* (green). Scale bars: 10 μm. All *P* values are listed in Supplementary Dataset 5. Source data are provided as a Source Data file.

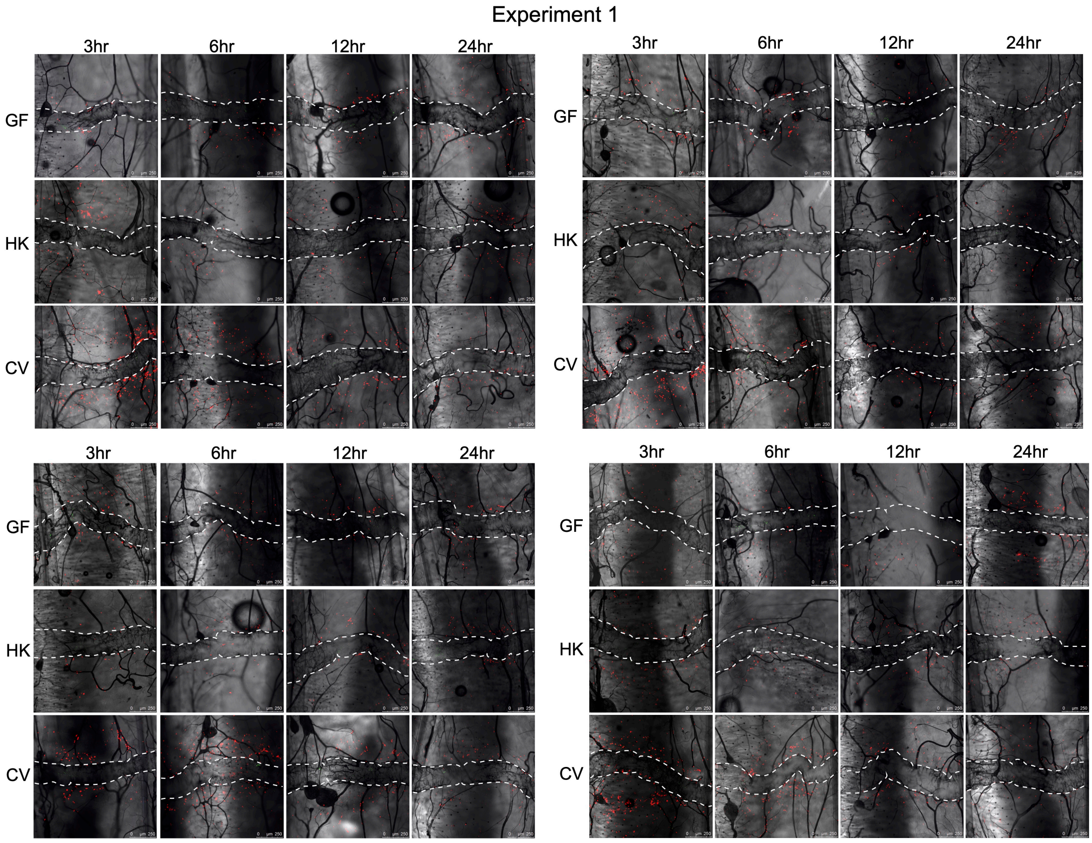

Supplementary Fig. 2 | Hemocytes aggregation post-GFP-*Hafnia* injection (green). Hemocytes were stained with CM-DiI (red). Dorsal vessels were outlined with white dotted lines. Scale bars: 250 μm. These are independent replicates of Fig. 1e.

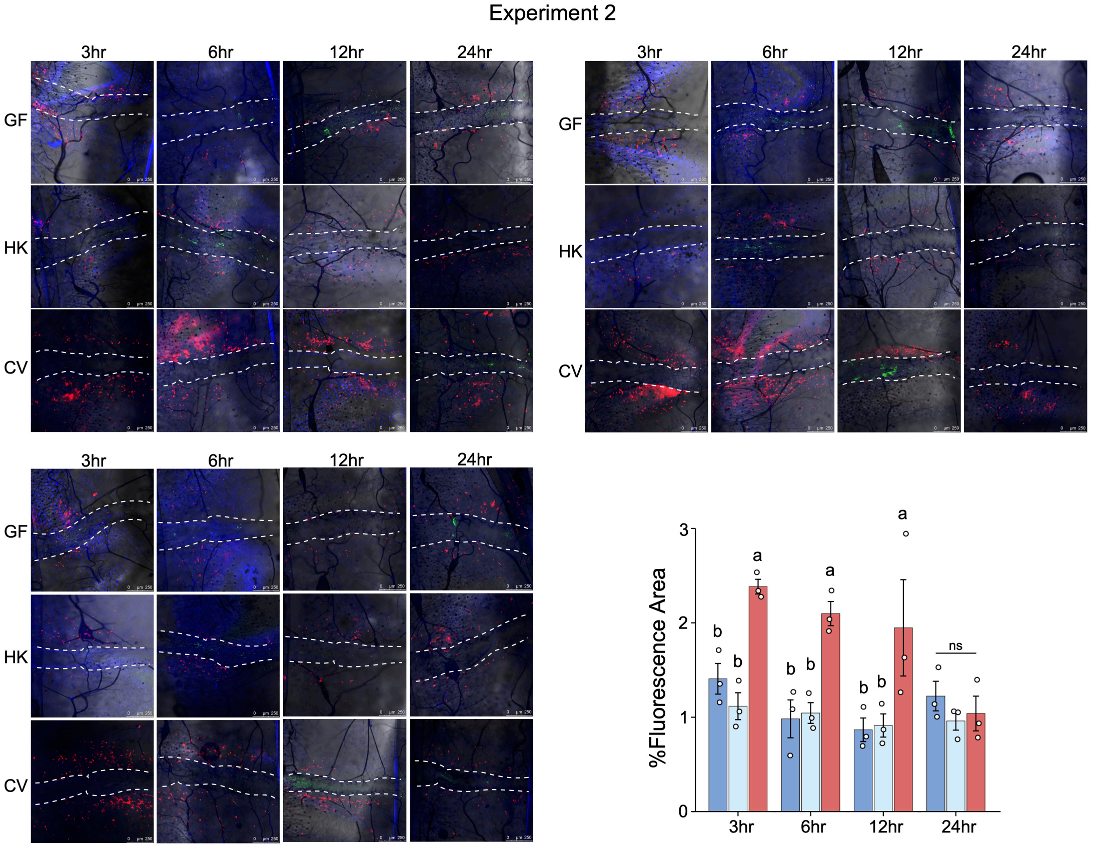

Supplementary Fig. 3 | Quantitative analysis of CM-Dil staining in hemocytes from experiment trial 2. Two-way ANOVA with the Tukey's multiple comparisons test. Different letters above bars indicate statistically significant differences among treatments (P < 0.05). ns: not significant. All P values are listed in Supplementary Dataset 5. Source data are provided as a Source Data file.

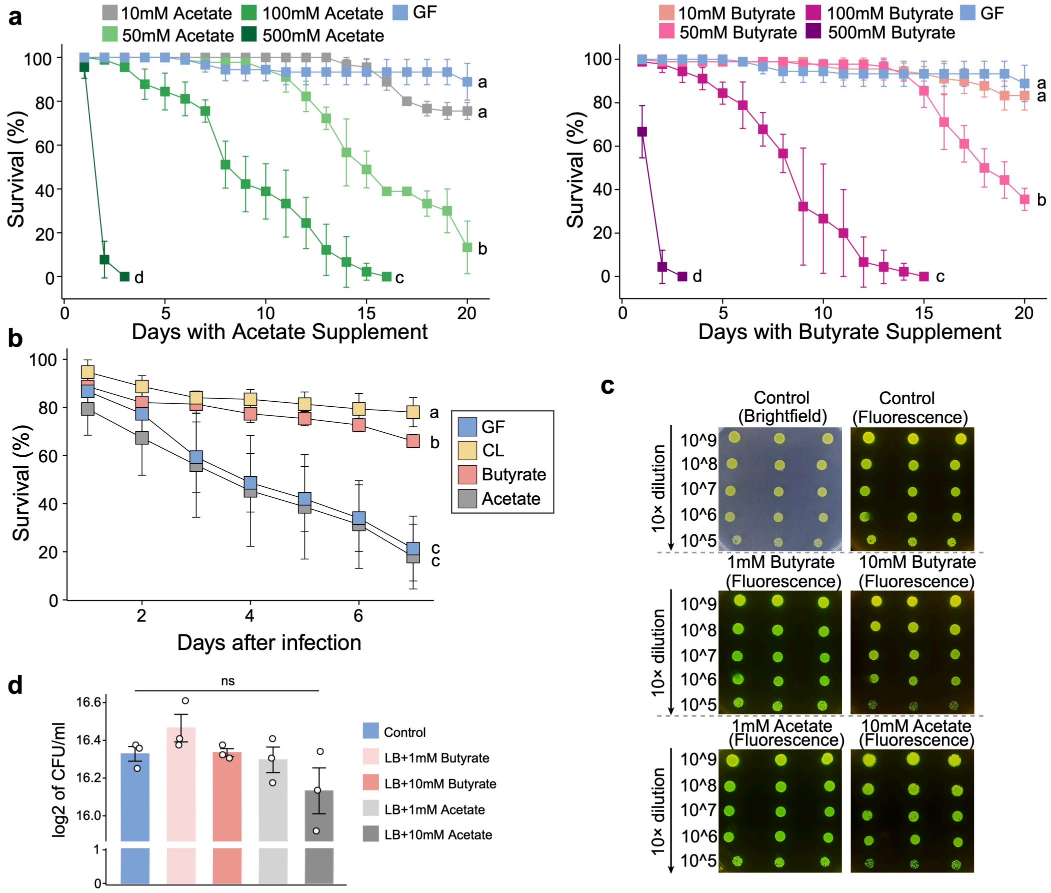

Supplementary Fig. 4 | The effects of acetate and butyrate supplementation on bee survival, and the impact of butyrate on *Hafnia* growth *in vitro*. a Survivorship of bees supplemented with gradient concentrations of acetate or butyrate (10 mM, 50 mM, 100 mM, and 500 mM). Bars represent mean ± s.d. n = 3 replicate for each group, with 30 individuals in each replicate. Experiment was repeated twice with similar results. **b** Survival rates of GF, CL (inoculated with a set of five core bacteria mixed at equal numbers), acetate-supplemented, and butyrate-supplemented bees following *Hafnia* injection. Bars represent mean ± s.d. from five biological replicates for each group (30 individuals per replicate). The results are independent replicates of Fig. 2a. (**a-b**) Two-sided Mantel-Cox test. Different letters

above bars indicate statistically significant differences among treatments ($P < 0.05$). (**c-d**) Abundance of GFP-*Hafnia* on media supplemented with butyrate or acetate. Bars represent mean ± s.e.m. Two-sided Student's t-test. ns: not significant. All P values are listed in Supplementary Dataset 5. Source data are provided as a Source Data file.

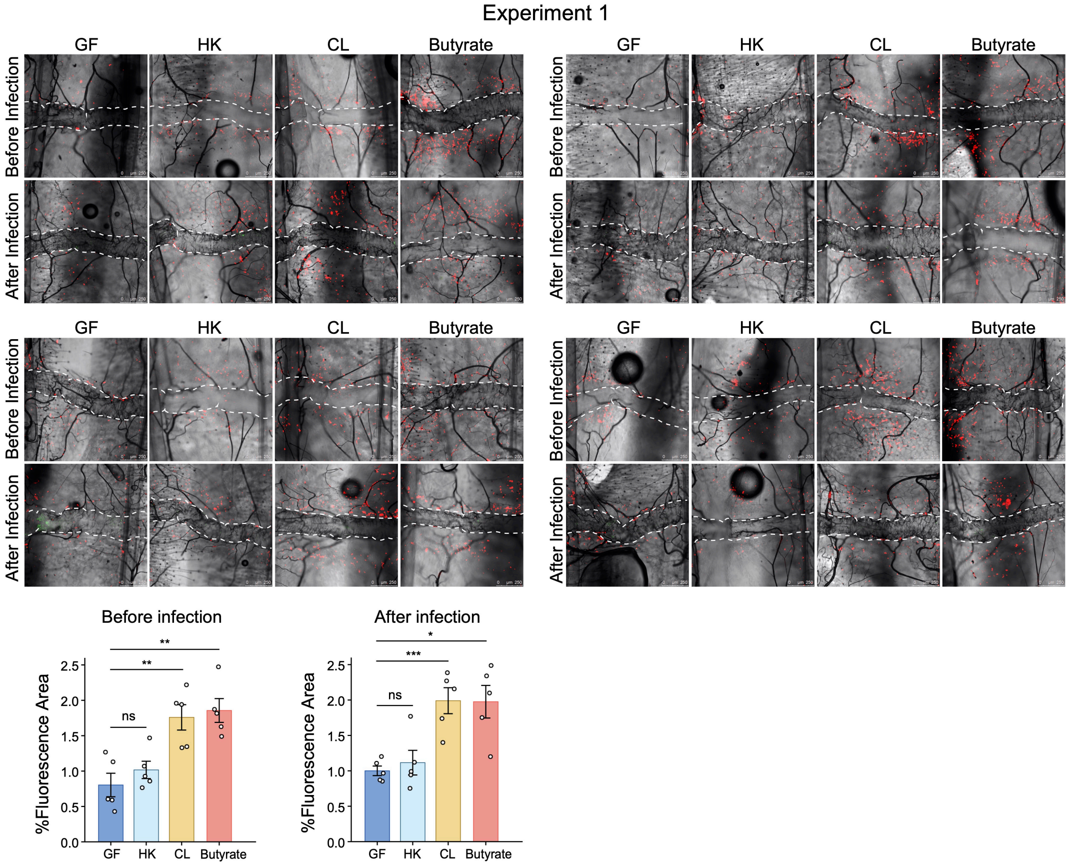

Supplementary Fig. 5 | CM-DiI-stained hemocytes (red) of GF, HK, CL, and butyrate-supplemented bees before *Hafnia* infection and at 3 hr post-GFP-*Hafnia* infection (green). Dorsal vessels were outlined with white dotted lines. Scale bars: 250 μm. These are independent replicates of Fig. 2d. Bars represent mean ±

s.e.m. Two-sided Student’s t-test. ns: not significant, *$P < 0.05$, **$P < 0.01$, ***$P < 0.001$. All P values are listed in Supplementary Dataset 5. Source data are provided as a Source Data file.

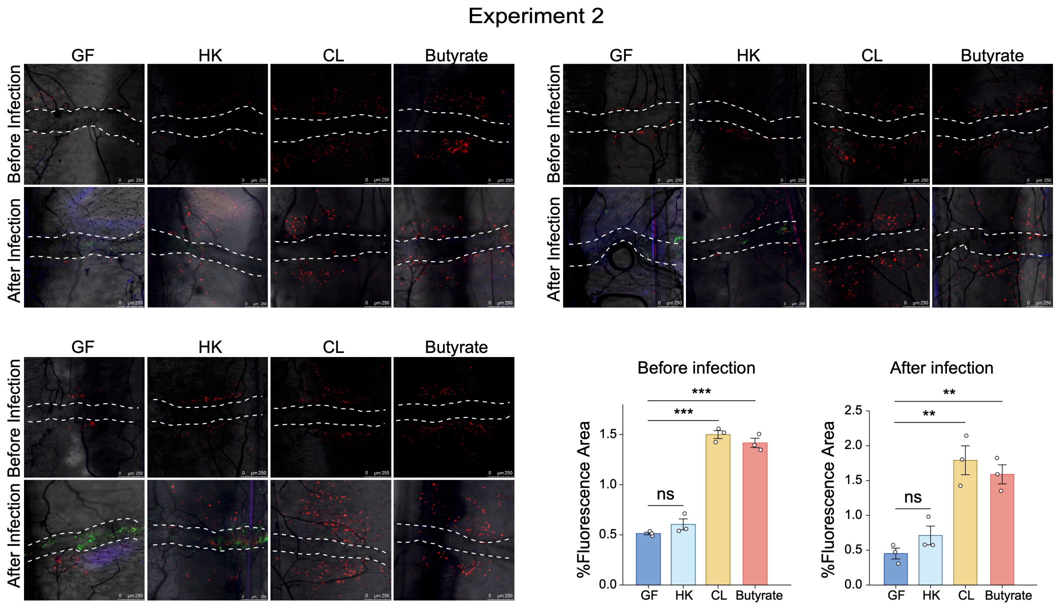

Supplementary Fig. 6 | Quantitative analysis of CM-Dil-stained hemocytes of GF, HK, CL, and butyrate-supplemented bees before *Hafnia* infection and at 3 hr post-GFP-*Hafnia* infection from experiment trial 2. Bars represent mean ± s.e.m. Two-sided Student’s t-test. ns: not significant, **$P < 0.01$, ***$P < 0.001$. All P values are listed in Supplementary Dataset 5. Source data are provided as a Source Data file.

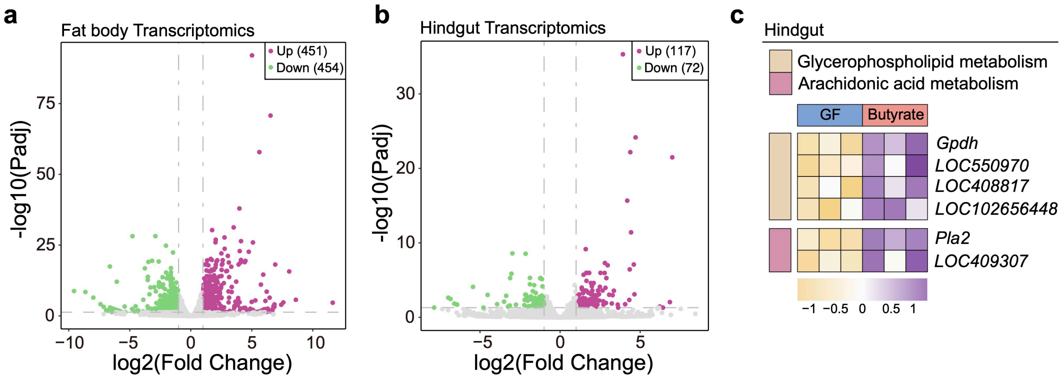

Supplementary Fig. 7 | Transcriptome analysis of honey bee fat bodies and hindguts, and Nile red staining in fat bodies. (**a-b**) Volcano plots showing differentially expressed genes (DEGs) in the fat bodies (**a**) and hindguts (**b**) of butyrate-supplemented bees compared to GF bees. **c** Heatmap of DEGs involved in glycerophospholipid and arachidonic acid metabolism in the hindguts of GF and butyrate-supplemented bees. Source data are provided as a Source Data file.

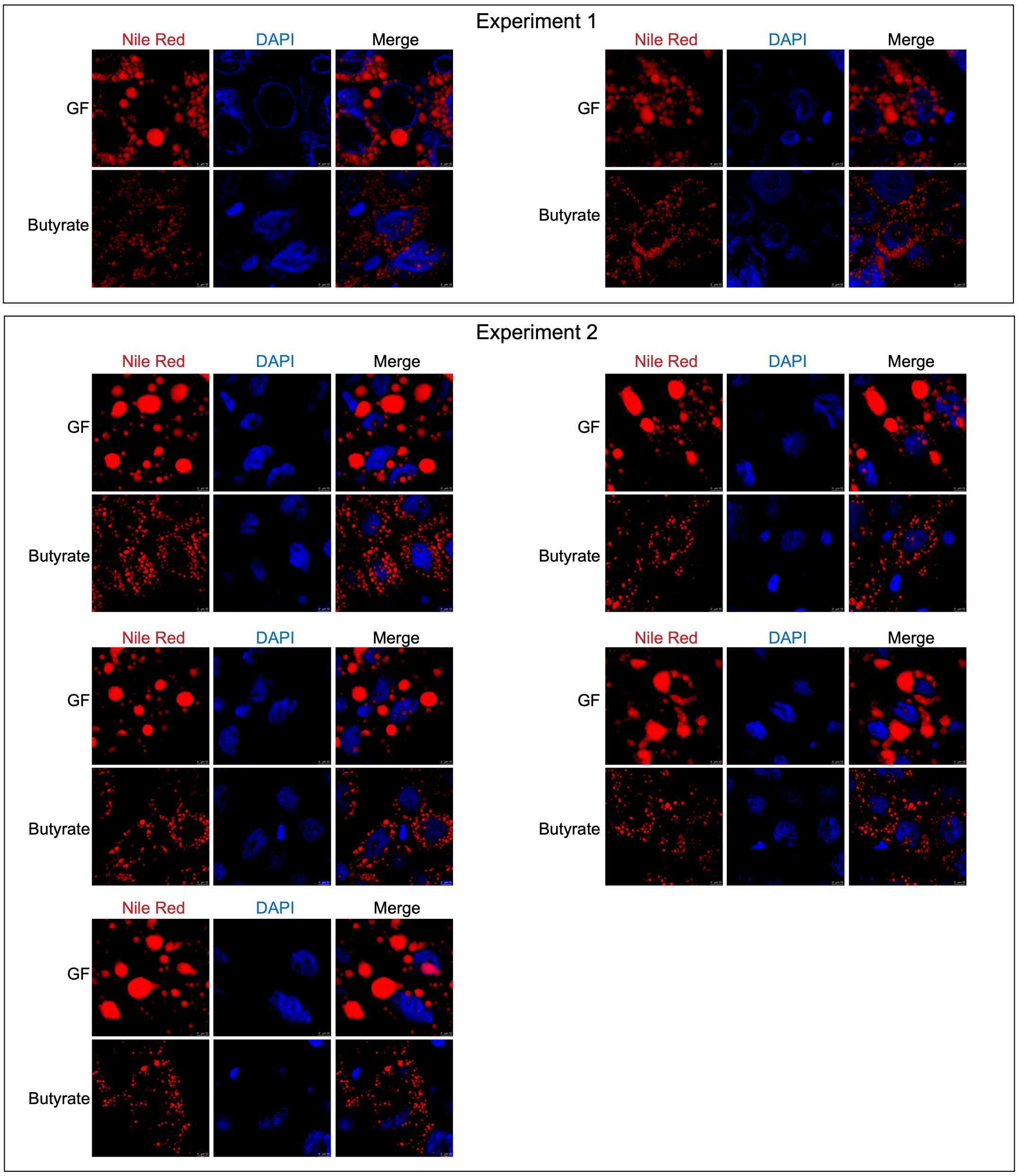

Supplementary Fig. 8 | Nile red staining in honey bee fat bodies. Nile red staining of neutral lipids (red) in the honey bee fat bodies on day 5 after butyrate supplementation, with cell nuclei stained with DAPI (blue). Images are from experiment trial 1 and 2. These are independent replicates of Fig. 3d.

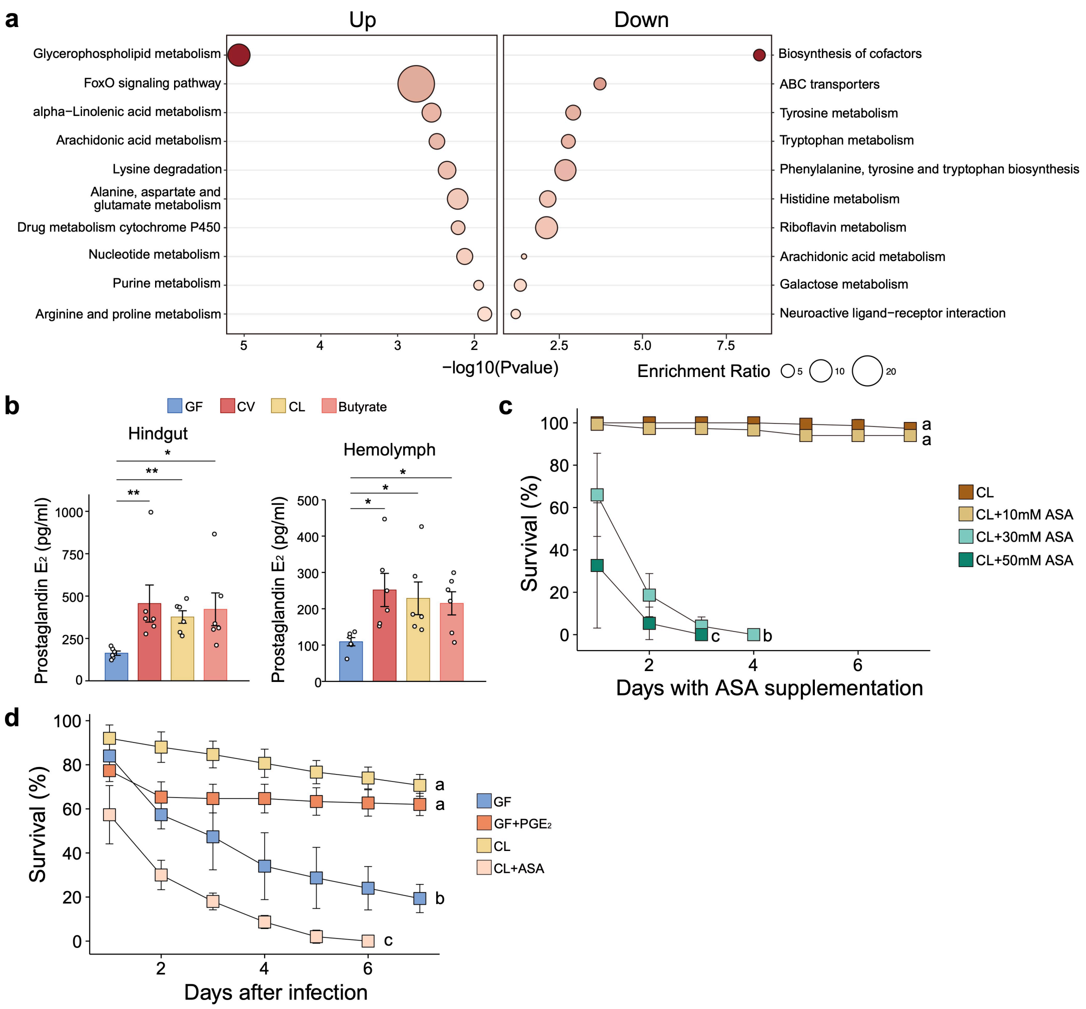

Supplementary Fig. 9 | Metabolic pathway analysis of the abdomens in GF and butyrate-supplemented bees, and honey bee prostaglandin production activates systemic cellular immune response. a Enriched KEGG pathways of discriminating metabolites in the abdomens of butyrate-supplemented bees compared to GF bees. One-sided hypergeometric test. **b** Prostaglandin E_2 (PGE_2) levels in the hindgut and hemolymph of GF, CV, CL, and butyrate-supplemented bees at day 5 post-colonization. Bars represent mean ± s.e.m. n = 6 replicates per group. Each replicate was pooled from 5 individuals from 3 cup cages. These are independent replicates of Fig. 4c. Two-sided Mann–Whitney test or two-sided

unpaired Student's t-test, depending on data normality. *$P < 0.05$, **$P < 0.01$. **c** Survival rates of CL bees continuously fed with different concentrations of acetylsalicylic acid (ASA; 10 mM, 30 mM, and 50 mM) starting from day 3 post-emergence, with CL bees as the control. Bars represent mean ± s.d. from five biological replicates (30 individuals per replicate). **d** Survival rates of GF, GF+PGE_2, CL, and CL+ASA bees post-*Hafnia* infection. Bars represent mean ± s.d. from five biological replicates for each group (30 individuals in each replicate). These are independent replicates of Fig. 4f. In (**c-d**), two-sided Mantel–Cox test. Different letters above bars indicate statistically significant differences among treatments ($P < 0.05$). All *P* values are listed in Supplementary Dataset 5. Source data are provided as a Source Data file.

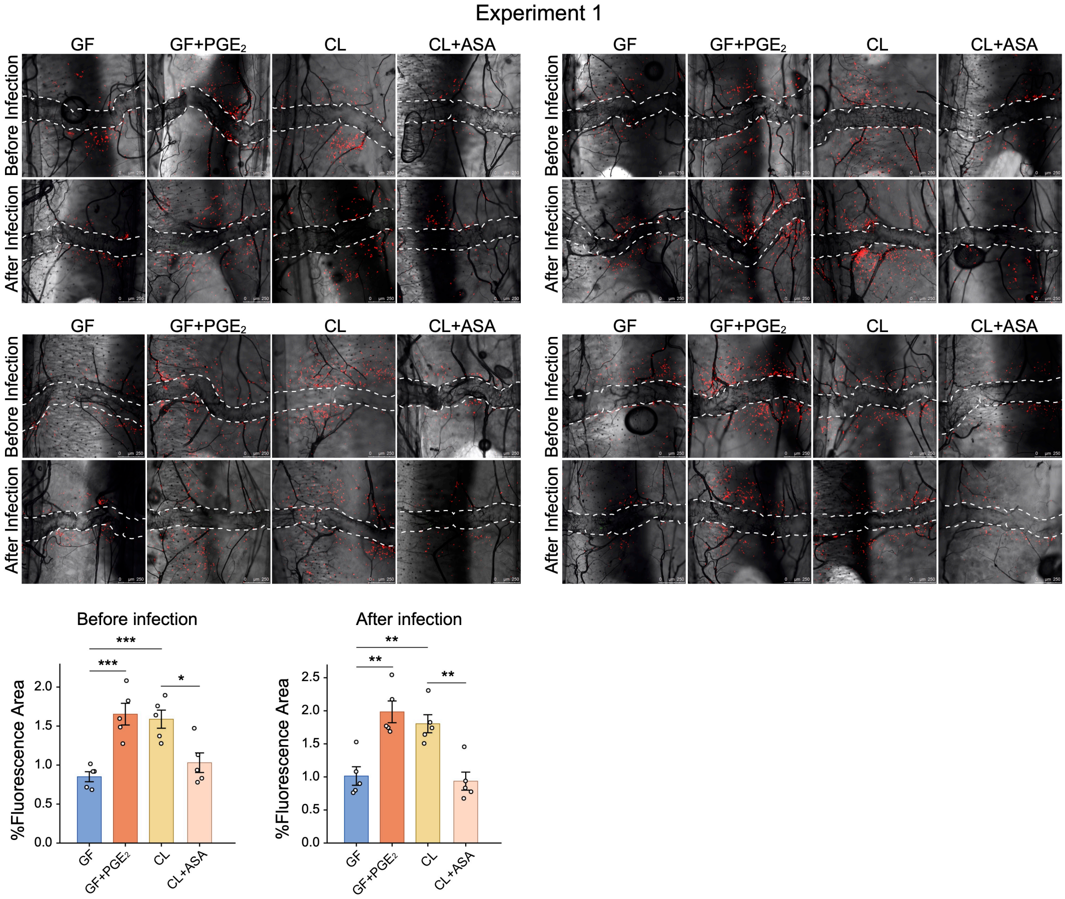

Supplementary Fig. 10 | Hemocytes stained with CM-DiI (red) in GF bees, GF bees supplemented with PGE_2, CL bees, and CL bees treated with ASA. Staining was performed before GFP-*Hafnia* infection (3 hr after PGE_2 supplementation) and at 3 hr post-infection with GFP-*Hafnia* (green). Dorsal vessels were outlined with white dotted lines. Scale bars: 250 μm. These are independent replicates of Fig. 4h. Two-sided Student's t-test. *$P < 0.05$, **$P < 0.01$, ***$P < 0.001$. All *P* values are listed in Supplementary Dataset 5. Source data are provided as a Source Data file.

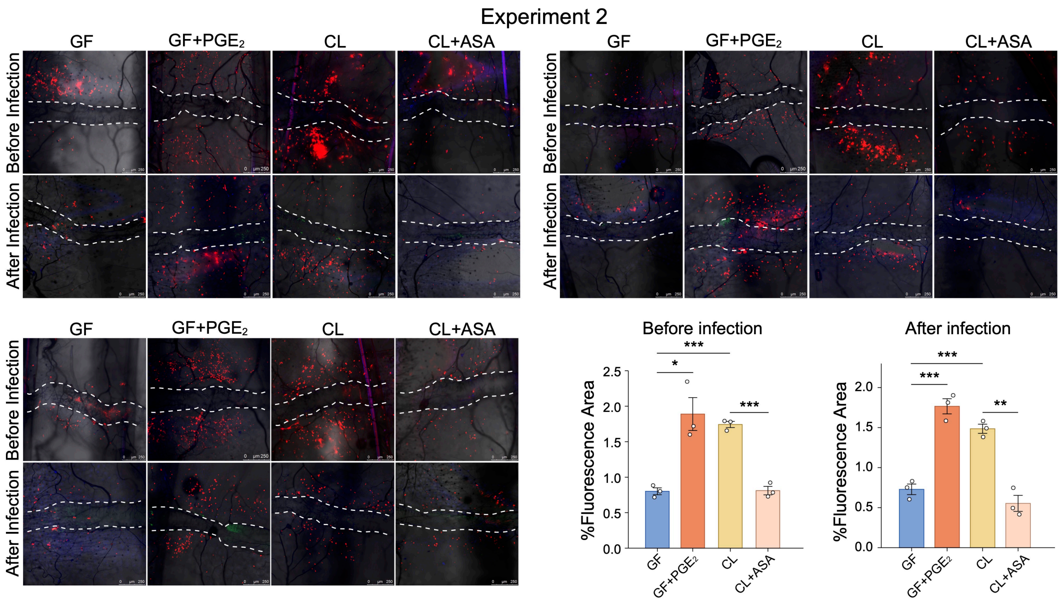

Supplementary Fig. 11 | Quantitative analysis of hemocytes stained with CM-DiI (red) in GF bees, GF bees supplemented with PGE_2, CL bees, and CL bees treated with ASA from experiment trail 2. Bars represent mean ± s.e.m. Two-sided Student's t-test. **P* < 0.05, ***P* < 0.01, ****P* < 0.001. All *P* values are listed in Supplementary Dataset 5. Source data are provided as a Source Data file.

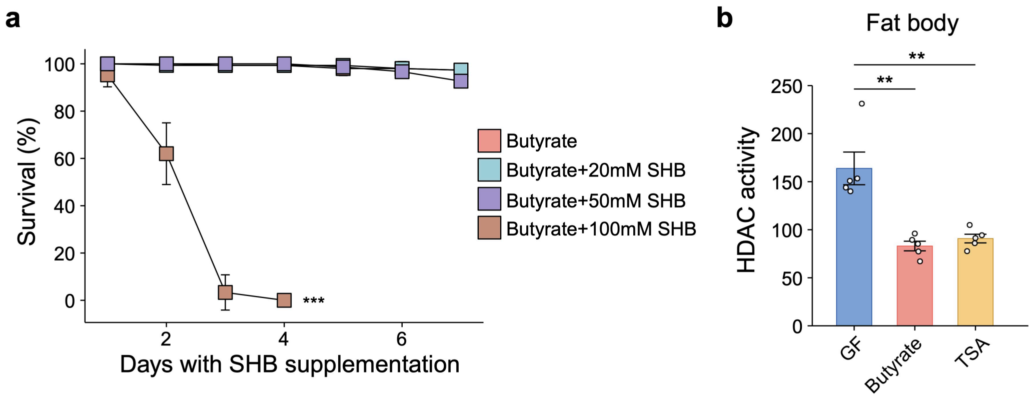

Supplementary Fig. 12 | Survival rates of butyrate-supplemented bees fed with β-hydroxybutyrate, and fat body HDAC activity. a Survival rates of butyrate-supplemented bees continuously fed with different concentrations of β-

hydroxybutyrate (SHB; 20 mM, 50 mM, and 100 mM). Butyrate-supplemented bees served as the control. Bars represent mean ± s.d. from five biological replicates (30 individuals per replicate). Two-sided Mantel–Cox test. ***P < 0.001. **b** Fat body HDAC activity in GF, butyrate-supplemented, and TSA-treated bees. Bars represent mean ± s.e.m. n = 5 replicates per group from one experiment. Each replicate was pooled from 5 individuals from 3 cup cages. These are independent replicates of Fig. 5c. Two-sided Mann–Whitney test. ***P* < 0.01. All *P* values are listed in Supplementary Dataset 5. Source data are provided as a Source Data file.

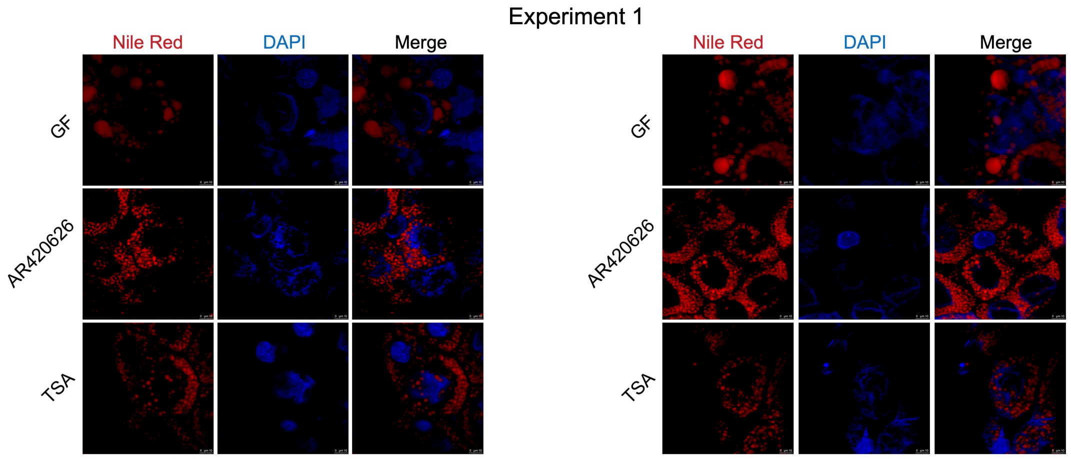

Supplementary Fig. 13 | Nile red staining of neutral lipids (red) in fat body of GF, AR420626- and TSA-treated bees. Cell nuclei are stained with DAPI (blue). These are independent replicates of Fig. 5e. Scale bars: 10 μm.

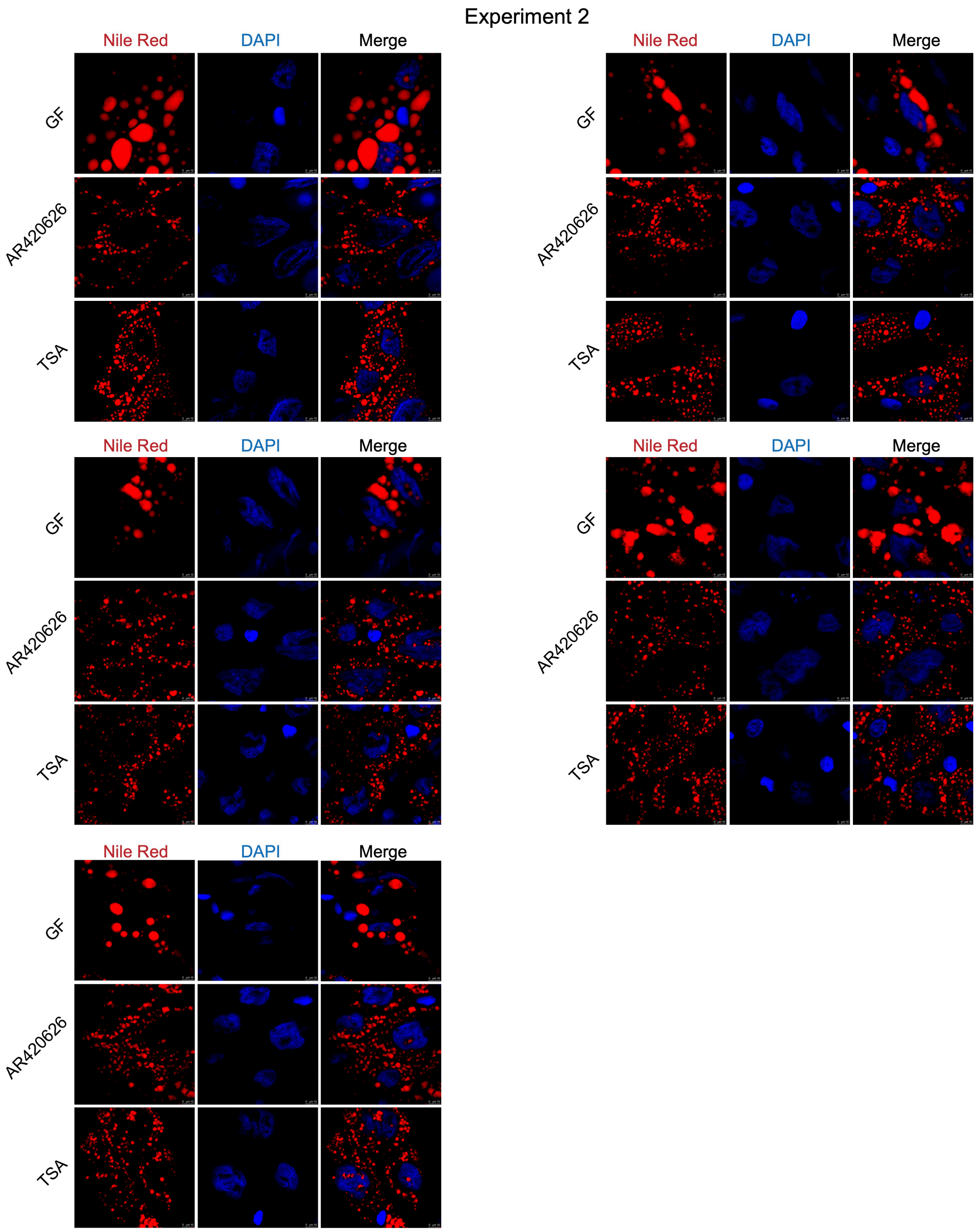

Supplementary Fig. 14. Nile red staining of neutral lipids (red) in fat body of GF, AR420626- and TSA-treated bees from experiment trial 2. Cell nuclei are stained with DAPI (blue). Scale bars: 10 μm.

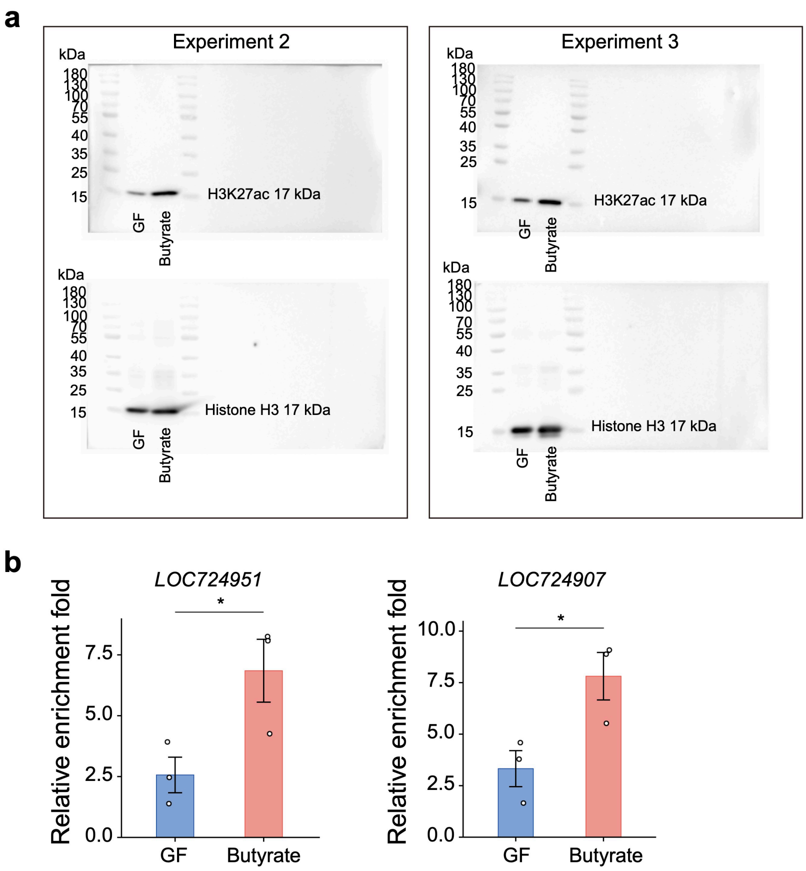

Supplementary Fig. 15. The effect of butyrate on H3K27ac modification and ChIP-qPCR. a Western blot analysis of H3K27ac levels in the fat bodies of GF and butyrate-supplemented bees. Total H3 antibody was used as a loading control. These are independent replicates of Fig. 5e. **b** ChIP-qPCR of H3K27ac at *LOC724951* and *LOC724907* promoters. Bars represent mean ± s.e.m. n = 3 replicates per group, with each replicate pooled from 5 individuals from 3 cup cages. These are independent replicates of Fig. 6h and 6i. *P* = 0.0477 (*LOC724951*) and 0.0362 (*LOC724907*) from two-sided Student’s t-test. Source data are provided as a Source Data file.

Supplementary Table 1. Bacterial strains used for colonization

Bacterial strain	Inoculum	Culture medium	Culture condition	Strain source
Bifidobacterium asteroides W8118	Ba and CL	TPY Agar Medium (Hopebiol, China)	35°C, microaerophilic	[1]
Gilliamella apicola W8127	Ga and CL	Brain Heart Infusion (BHI) Agar Medium (Thermo Fisher Scientific, USA)	35°C, microaerophilic	[1,2]
Snodgresella alvi H11	Sa and CL	Brain Heart Infusion (BHI) Agar Medium (Thermo Fisher Scientific, USA)	35°C, microaerophilic	[1]
Bombilactobacillus W8086	F4 and CL	MRS Agar Medium (Hopebiol, China)	34°C, microaerophilic	[1]
Lactobacillus W8171	F5 and CL	MRS Agar Medium (Hopebiol, China)	34°C, microaerophilic	[1]

Supplementary Table 2. Primers used in this study.

Gene	NCBI sequence accessions	Sequence (5'-3')	Efficiency	Source
Universal bacteria (*16S rRNA*)		**F:** AGGATTAGATACCCTGGTAGTCC<br>**R:** YCGTACTCCCCAGGCGG		3
Actin	NM_001185146.1	**F:** TGCCAACACTGTCCTTTCTG<br>**R:** AGAATTGACCCACCAATCCA		4
Apidaecin	NM_001011642.1	**F:** GTAGGTCGAGTAGGCGGATCT<br>**R:** TTTTGCCTTAGCAATTCTTGTTG		5
Abaecin	NM_001011617.1	**F:** TCGGATTGAATGGTCCCTGAC<br>**R:** ATCTTCGCACTACTCGCCAC		5
Defensin-1	NM_001011616.2	**F:** TGCGCTGCTAACTGTCTCAG<br>**R:** AATGGCACTTAACCGAAACG		6
Hymenoptaecin	NM_001011615.1	**F:** GTCGTCCATCCTTGGACATT<br>**R:** TTTCCCAAACTCGAATCCTG		7
Toll	XM_026440067.1	**F:** TAGAGTGGCGCATTGTCAAG<br>**R:** ATCGCAATTTGTCCCAAAAC		8
Cactus-1	XM_006567107.2	**F:** CTATCGTGGAGAAACTGCGTAT<br>**R:** TCAGGAAGTGGTTCTGGTATTG		9
Cactus-2	XM_394485.7	**F:** ATCAGACGGCTCTGCTCTAT<br>**R:** TCGTCTTCGTCAGTGGTATCT		9
Dorsal	XM_006566997.3	**F:** AGAGATGGAACGCAGGAAAC<br>**R:** TGACAGGATATAGGACGAGGTAA		8
PGRP-LC	XM_006565506.3	**F:** TCCGTCAGCCGTAGTTTTTC<br>**R:** CGTTTGTGCAAATCGAACAT		8
Dredd	XM_006570913.3	**F:** GCGTCATAAAGAAAAAGGATCA<br>**R:** TTTCGGGTAATTGAGCAACG		8
Relish	XM_026444179.1	**F:** GGAGCTGATCCAAATCGAAC<br>**R:** AGTGGCATCCATCCATCATT		7
LOC408559	XM_392104.6	**F:** ATGGTCTTGCTGCTGGAGTT<br>**R:** TCCTTCTGCACCGAGTTCAC	1.09	This study
LOC551968	XM_624350.5	**F:** TCCTAGTTTTAGCGATGCAGACT<br>**R:** TGGGTGACATTCAATTTGGTTTG	0.97	This study
LOC409261	XM_026445108.1	**F:** AAACCGGTCGCAAAACAAGT<br>**R:** TGCTTCGCATTGAATACCACAA	1.01	This study
mino	XM_395192.7	**F:** ACCGTGGTATTGCCATCTCAAA<br>**R:** TCGTAAGAGCCACCCAAACA	0.98	This study
LOC724951	XM_001120852.5	**F**: AGAGATTTGATTCTGGTCGAGTT<br>**R**: GTCAAGCGTTAATCCTTCCGT	1	This study
LOC724995	XM_001120897.5	**F:** AAGAAATTTGATTCTGGTCGAGTT<br>**R:** AGTTCACGTAAAGTCTCGCTCA	1.04	This study
Lpin	XM_393684.7	**F:** CGCAAACATTCCAATCATCATATTC<br>**R:** CCGGTATTGGATCTCGCCAA	0.94	This study
LOC726880	XM_026442631.1	**F:** AAGCCGTGGCCAATACAAGA<br>**R:** CAGCAGGATCAAGGCCAGTAA	0.92	This study
bbc	XM_016916632.2	**F:** GACAGTAATTTGTGCAGTGGCA<br>**R:** AAACTGATGTTCCAGCCACA	1	This study
Pla2	NM_001011614.1	**F:** TGAGAGAACCGAGGGTCGTT<br>**R:** TCAATACTTGCGAAGATCGAACC	0.91	This study
dsGFP (Primers for		**F:**<br>TAATACGACTCACTATAGGGCGAACG		This study

Gene	NCBI sequence accessions	Sequence (5'-3')	Efficiency	Source
dsRNA)		GGTAAACTACCT<br>**R:**<br>TAATACGACTCACTATAGGGCGATTCTTTTGCTTGTCGGC		
dsPla2 (Primers for dsRNA)		**F:**<br>TAATACGACTCACTATAGGGATGCAAGTCGTTCTCGGATCC<br>**R:**<br>TAATACGACTCACTATAGGGGTGACAGGATGCTCCAGTTT		This study
DNA-spikein (Primers for ChIP)		**F:** GCCTTCTTCCCATTTCTGATCC<br>**R:** CACGAATCAGCGGTAAAGGT		This study
LOC724951 (Primers for ChIP)		**F:** ACCGAATTTTGTATAGAAGCGAAA<br>**R:** AGTGCGATACAACGTATTAGTGT		This study
LOC724907 (Primers for ChIP)		**F:** ATGGTCACAAAGATGGTCATGC<br>**R:**<br>GTTTGTTACTCACTGTTGAGACTGT		This study

References

1. Han, B. *et al. Lactobacillus* Firm-5-derived succinate prevents honeybees from having diabetes-like symptoms. *Proc. Natl. Acad. Sci. U. S. A.* **121**, e2405410121 (2024).
2. Zheng, H. *et al.* Division of labor in honey bee gut microbiota for plant polysaccharide digestion. *Proc. Natl. Acad. Sci. U. S. A.* **116**, 25909–25916 (2019).
3. Kešnerová, L. *et al.* Disentangling metabolic functions of bacteria in the honey bee gut. *PLoS Biol* **15**, e2003467 (2017).
4. Zufelato, M. S., Lourenço, A. P., Simões, Z. L. P., Jorge, J. A. & Bitondi, M. M. G. Phenoloxidase activity in *Apis mellifera* honey bee pupae, and ecdysteroid-dependent expression of the prophenoloxidase mRNA. *Insect Biochem. Mol. Biol.* **34**, 1257–1268 (2004).
5. Emery, O., Schmidt, K. & Engel, P. Immune system stimulation by the gut symbiont *Frischella perrara* in the honey bee (*Apis mellifera*). *Mol. Ecol.* **26**, 2576–2590 (2017).
6. Evans, J. D. Transcriptional immune responses by honey bee larvae during invasion by the bacterial pathogen, *Paenibacillus larvae*. *J. Invertebr. Pathol.* **85**, 105–111 (2004).
7. Lourenço, A. P., Guidugli-Lazzarini, K. R., Freitas, F. C. P., Bitondi, M. M. G. & Simões, Z. L. P. Bacterial infection activates the immune system response and dysregulates microRNA expression in honey bees. *Insect Biochem. Mol. Biol.* **43**, 474–482 (2013).
8. Tesovnik, T. *et al.* Immune related gene expression in worker honey bee (*Apis mellifera carnica*) pupae exposed to neonicotinoid thiamethoxam and *Varroa* mites (*Varroa destructor*). *PLoS One* **12**, e0187079 (2017).
9. Leonard, S. P. *et al.* Engineered symbionts activate honey bee immunity and limit pathogens. *Science* **367**, 573–576 (2020).
