# Supplementary material for: Gut microbiota-derived butyrate primes systemic immunity in honey bees by mediating lipid metabolic reprogramming
Source: Nat Commun. 2026 Feb 2;17:2924. doi: 10.1038/s41467-026-69073-0 (PMC13031938; doi:10.1038/s41467-026-69073-0)
Supplement: Supplementary file 2 — Description of Additional Supplementary Files [file 41467_2026_69073_MOESM2_ESM.pdf]

Title: Supplementary Data 1

Description: The fold changes (Butyrate/GF) of differentially expressed genes (DEGs) of honey bee fat body 5 days after butyrate supplementation.

Title: Supplementary Data 2

Description: The fold changes (Butyrate/GF) of differentially expressed genes (DEGs) of honey bee hindgut 5 days after butyrate supplementation.

Title: Supplementary Data 3

Description: The fold changes (Butyrate/GF) and abundance (peak heights for the quantification at the specific retention index) of metabolites of honey bee abdomen 5 days after butyrate supplementation. 5

Title: Supplementary Data 4

Description: qPCR data

Title: Supplementary Data 5

Description: Exact P value
