# Supplementary material for: Gut microbiota-derived butyrate primes systemic immunity in honey bees by mediating lipid metabolic reprogramming
Source: Nat Commun. 2026 Feb 2;17:2924. doi: 10.1038/s41467-026-69073-0 (PMC13031938; doi:10.1038/s41467-026-69073-0)
Supplement: Supplementary file 8 — Reporting summary [file 41467_2026_69073_MOESM8_ESM.pdf]

nature portfolio

Corresponding author(s): Dr Shiqi Luo

Last updated by author(s): 26/01/26

Reporting Summary

Nature Portfolio wishes to improve the reproducibility of the work that we publish. This form provides structure for consistency and transparency in reporting. For further information on Nature Portfolio policies, see our Editorial Policies and the Editorial Policy Checklist.

Statistics

For all statistical analyses, confirm that the following items are present in the figure legend, table legend, main text, or Methods section.

n/a	Confirmed	
☐	☒	The exact sample size (n) for each experimental group/condition, given as a discrete number and unit of measurement
☐	☒	A statement on whether measurements were taken from distinct samples or whether the same sample was measured repeatedly
☐	☒	The statistical test(s) used AND whether they are one- or two-sided<br>*Only common tests should be described solely by name; describe more complex techniques in the Methods section.*
☒	☐	A description of all covariates tested
☐	☒	A description of any assumptions or corrections, such as tests of normality and adjustment for multiple comparisons
☐	☒	A full description of the statistical parameters including central tendency (e.g. means) or other basic estimates (e.g. regression coefficient) AND variation (e.g. standard deviation) or associated estimates of uncertainty (e.g. confidence intervals)
☐	☒	For null hypothesis testing, the test statistic (e.g. F, t, r) with confidence intervals, effect sizes, degrees of freedom and P value noted<br>*Give P values as exact values whenever suitable.*
☒	☐	For Bayesian analysis, information on the choice of priors and Markov chain Monte Carlo settings
☒	☐	For hierarchical and complex designs, identification of the appropriate level for tests and full reporting of outcomes
☒	☐	Estimates of effect sizes (e.g. Cohen's d, Pearson's r), indicating how they were calculated

Our web collection on statistics for biologists contains articles on many of the points above.

Software and code

Policy information about availability of computer code

Data collection

For RNA-seq, sequencing libraries were prepared using the NEBNext Ultra RNA Library Prep Kit for Illumina (NEB, USA) and PCR products were purified with the AMPure XP system. Library quality was assessed with the Agilent 5400 system (Agilent, USA). Sequencing was performed on the Illumina NovaSeq platform producing 150 bp paired-end reads. Raw reads with quality score below 20 in more than 10% of bases were discarded using fastp (version 0.13.1). For CUT&Tag sequencing, DNA library construction and quality assessment were performed by Novogene. Library concentration was measured using Qubit, fragment integrity was assessed using AATI, and effective library concentration was determined by qPCR. Sequencing was conducted on the Illumina NovaSeq platform (PE150). Raw reads were quality-filtered using Trim Galore(v0.6.10).

Data analysis

Clean RNA-seq reads were quality-filtered using fastp (version 0.13.1). Reads were mapped to the Apis mellifera reference genome (Amel_HAv3.1) using HISAT2 (version 2.1.0), and transcripts were assembled with StringTie (version 2.0.6). Differential gene expression analysis was performed in R (version 4.2.3) using the DESeq2 package (version 1.38.3), with significantly differentially expressed genes defined as adjusted p-value < 0.05 and |log2-fold change| ≥ 1. Pathway enrichment analysis of DEGs was conducted using the R package ClusterProfiler(version 4.6.2). CUT&Tag raw reads were quality-filtered using Trim Galore(version 0.6.10), aligned to the Apis mellifera reference genome using Bowtie 2(version 2.3.4.3), and duplicate/unmapped reads were removed with Sambamba(version 0.6.6) and Samtools(version 1.9). Enriched peaks were called with MACS2(version 2.2.6), and peak annotation was performed using the R package ChIPseeker (version 1.43.0). Differential peak analysis was conducted in R using DiffBind (version 3.8.4, p-value < 0.05, Fold change > 1.5), and KEGG enrichment analysis of differential peaks was performed using the R package ClusterProfiler (version 4.6.2). Relevant codes were uploaded to Github and archived in Zenodo under DOI: 10.5281/zenodo.18043934, and Figshare (https://doi.org/10.6084/m9.figshare.30403936) for public access. All other data analyses were conducted using GraphPad Prism (version 9.3.1).

For manuscripts utilizing custom algorithms or software that are central to the research but not yet described in published literature, software must be made available to editors and reviewers. We strongly encourage code deposition in a community repository (e.g. GitHub). See the Nature Portfolio guidelines for submitting code & software for further information.

Data

Policy information about availability of data

All manuscripts must include a data availability statement. This statement should provide the following information, where applicable:

- Accession codes, unique identifiers, or web links for publicly available datasets
- A description of any restrictions on data availability
- For clinical datasets or third party data, please ensure that the statement adheres to our policy

The sequencing data were uploaded to NCBI Bioproject with an accession number PRJNA1232651 and PRJNA1233551. The metabolomic raw data were uploaded to Metabolights with accession number MTBLS13098.

Research involving human participants, their data, or biological material

Policy information about studies with human participants or human data. See also policy information about sex, gender (identity/presentation), and sexual orientation and race, ethnicity and racism.

Reporting on sex and gender	No human participants were involved in this study.
Reporting on race, ethnicity, or other socially relevant groupings	No human participants were involved in this study.
Population characteristics	No human participants were involved in this study.
Recruitment	No human participants were involved in this study.
Ethics oversight	No human participants were involved in this study.

Note that full information on the approval of the study protocol must also be provided in the manuscript.

Field-specific reporting

Please select the one below that is the best fit for your research. If you are not sure, read the appropriate sections before making your selection.

☒ Life sciences ☐ Behavioural & social sciences ☐ Ecological, evolutionary & environmental sciences

For a reference copy of the document with all sections, see nature.com/documents/nr-reporting-summary-flat.pdf

Life sciences study design

All studies must disclose on these points even when the disclosure is negative.

Sample size	All experiments were performed with 5–10 independent biological replicates per group, or 2–3 independent experimental batches, depending on the experiment.
Data exclusions	No data were excluded from the analyses.
Replication	All experiments were performed with 5–10 independent biological replicates per group, or 2–3 independent experimental batches, depending on the experiment. All attempts at replication were successful.
Randomization	Dark-eyed pupae of Apis mellifera were removed from capped brood cells using sterile tweezers and reared in sterile plastic cups under controlled conditions (35°C, 80% humidity) until eclosion. Twenty newly emerged bees under 24-hr age were randomly placed into each sterile plastic cup for bacterial inoculation.
Blinding	Blinding was not performed because the experimental treatments (GF or Butyrate) were applied in a controlled laboratory setting, and investigators could distinguish groups during handling. All downstream assays, including RNA-seq, CUT&Tag, metabolomics, qPCR, and confocal imaging, are objective measurements, so the lack of blinding is unlikely to have affected the results.

Reporting for specific materials, systems and methods

We require information from authors about some types of materials, experimental systems and methods used in many studies. Here, indicate whether each material, system or method listed is relevant to your study. If you are not sure if a list item applies to your research, read the appropriate section before selecting a response.

Materials & experimental systems

n/a	Involved in the study	
☐	☒	Antibodies
☒	☐	Eukaryotic cell lines
☒	☐	Palaeontology and archaeology
☐	☒	Animals and other organisms
☒	☐	Clinical data
☒	☐	Dual use research of concern
☒	☐	Plants

Methods

n/a	Involved in the study	
☐	☒	ChIP-seq
☒	☐	Flow cytometry
☒	☐	MRI-based neuroimaging

Antibodies

Antibodies used	Acetyl-Histone H3 (Lys27) (D5E4) XP® Rabbit mAb (Cat#8173, Cell Signaling Technology, USA) , Anti-Histone H3 Mouse Monoclonal Antibody (Cat#BE3015, Easybio, China), HRP-conjugated Goat Anti-Rabbit IgG (H+L) (Cat#SA00001-2, Proteintech, China) , HRP-conjugated Goat Anti-Mouse IgG (H+L) (Cat#SA00001-1, Proteintech, China)
Validation	Primary antibody: Acetyl-Histone H3 (Lys27) (D5E4) XP® Rabbit mAb (Cat#8173, Cell Signaling Technology, USA). Specificity: Recognizes H3 acetylated at Lys27; no cross-reactivity with H3 acetylated at Lys9, 14, 18, 23, or 56; minor cross-reactivity with H2B Lys5. Species Reactivity: Validated for human, mouse, rat, monkey; predicted to react with hamster, Xenopus, zebrafish, horse, guinea pig, rabbit. Purification: Monoclonal antibody produced by immunizing animals with a synthetic peptide corresponding to residues surrounding acetylated Lys27 of human histone H3. Primary antibody: Histone H3 (Cat#BE3015, Easybio, China). Specificity: Detects endogenous Histone H3 protein. Species Reactivity: Validated for human, rat, mouse, zebrafish. Secondary antibody: HRP-conjugated Goat Anti-Rabbit IgG (H+L)(Cat#SA00001-2, Proteintech, China). Specificity: Recognizes rabbit IgG. Purification: Polyclonal; purified from antisera by immunoaffinity chromatography using antigens coupled to agarose beads. Species Reactivity: Rabbit. Secondary antibody: HRP-conjugated Goat Anti-Mouse IgG (H+L)(Cat#SA00001-1, Proteintech, China). Specificity: Recognizes mouse IgG. Purification: Polyclonal; purified from antisera by immunoaffinity chromatography using antigens coupled to agarose beads. Species Reactivity: Mouse.

Animals and other research organisms

Policy information about studies involving animals; ARRIVE guidelines recommended for reporting animal research, and Sex and Gender in Research

Laboratory animals	The honey bees (Apis mellifera) used in this study were obtained from an apiary in Changping, Beijing, China (40.1°N, 116.1°E). Within each experiment, bees for all treatment and control groups were derived from a single brood comb to control for genetic background.
Wild animals	The study did not involve wild animals.
Reporting on sex	All experimental animals were worker honey bees, which are female. Sex was not a variable in this study and no sex-based analyses were performed.
Field-collected samples	The study did not involve samples collected from the field.
Ethics oversight	There is no current requirement regarding insectcare and use in research.

Note that full information on the approval of the study protocol must also be provided in the manuscript.

Plants

Seed stocks	This study did not involve any plants.
Novel plant genotypes	This study did not involve any plants.
Authentication	This study did not involve any plants.

ChIP-seq

Data deposition

- [x] Confirm that both raw and final processed data have been deposited in a public database such as GEO.
- [x] Confirm that you have deposited or provided access to graph files (e.g. BED files) for the called peaks.

Data access links<br>*May remain private before publication.*	The CUT&Tag sequencing data were uploaded to NCBI Bioproject with an accession number and PRJNA1233551.
Files in database submission	GF-1_1.fq.gz, GF-1_2.fq.gz, GF-2_1.fq.gz, GF-2_2.fq.gz, GF-3_1.fq.gz, GF-3_2.fq.gz, Butyrate-1_1.fq.gz, Butyrate-1_2.fq.gz, Butyrate-2_1.fq.gz, Butyrate-2_2.fq.gz, Butyrate-3_1.fq.gz, Butyrate-3_2.fq.gz, IgG_1.fq.gz, IgG_2.fq.gz
Genome browser session<br>(e.g. UCSC)	https://genome.ucsc.edu/s/liujiaming/CUTTagGFButyrate<br>The bigwig files have been deposited on GitHub and archived in Zenodo under DOI: 10.5281/zenodo.18043934, and Figshare (https://doi.org/10.6084/m9.figshare.30403936).

Methodology

Replicates	Five carcasses were pooled as one biological replicate, with three biological replicates per group. IgG was used as a negative control.
Sequencing depth	CUT&Tag libraries were sequenced on the Illumina NovaSeq platform using paired-end 150 bp reads, with an average sequencing depth of approximately 6 Gb per sample. Sequencing and mapping statistics were as follows:<br>Butyrate-1: 37,143,349 total reads, 35,385,672 uniquely mapped reads (95.27%);<br>Butyrate-2: 36,691,946 total reads, 35,104,145 uniquely mapped reads (95.67%);<br>Butyrate-3: 31,629,826 total reads, 30,247,894 uniquely mapped reads (95.63%);<br>GF-1: 35,856,418 total reads, 34,389,013 uniquely mapped reads (95.91%);<br>GF-2: 34,124,511 total reads, 32,760,787 uniquely mapped reads (96.00%);<br>GF-3: 37,582,914 total reads, 36,113,723 uniquely mapped reads (96.09%);<br>IgG control: 25,902,008 total reads, 21,131,132 uniquely mapped reads (81.58%).
Antibodies	The primary antibodies used were Acetyl-Histone H3 (Lys27) (D5E4) XP® Rabbit mAb (Cat#8173, Cell Signaling Technology) and Rabbit IgG (Cat#A7016, Beyotime) as a negative control. The secondary antibody used was Goat Anti-Rabbit IgG H&L (Cat#Ab207-01, Vazyme, China).
Peak calling parameters	CUT&Tag reads were aligned to the Apis mellifera reference genome (Amel_HAv3.1) using Bowtie2 (v2.3.4.3) with parameters --very-sensitive -X 700 -x $bowtie2_index -p 15. The resulting SAM files were converted to sorted BAM files using Samtools (v1.9). PCR duplicates and low-quality or multi-mapped reads were removed using Sambamba (v0.6.6, -r -t 5) and Samtools (-F 1804 -f 2 -q 30). Peaks were identified using MACS2 (v2.2.6) with parameters -t -c -f BAMPE -g 2.239e8 -q 0.05 -B. GF and Butyrate group samples were compared against the IgG control to remove non-specific background signals.
Data quality	Raw paired-end CUT&Tag reads were processed using Trim Galore to remove adapters and low-quality bases. Clean reads were aligned to the Apis mellifera reference genome (Amel_HAv3.1) using Bowtie2 (--very-sensitive -X 700). PCR duplicates and low-quality or unmapped reads were removed using Sambamba and Samtools to retain high-quality, uniquely mapped reads. Peaks were called using MACS2 with IgG as a control (-f BAMPE -g 2.239e8 -B -q 0.05), where q-value ≤ 0.05 was used to define significant peaks. Differential peak analysis between GF and Butyrate groups was performed using the R package DiffBind, with significant peaks defined as |fold change| > 1.5 and p-value < 0.05. Peak annotation was performed using ChIPseeker, and KEGG enrichment analysis was conducted with ClusterProfiler.
Software	Trim Galore (v0.6.10) was used for adapter and quality trimming. Bowtie2 (v2.3.4.3) was used for read alignment to the Apis mellifera reference genome (Amel_HAv3.1). Sambamba (v0.6.6) and Samtools (v1.9) were used for PCR duplicate removal and filtering low-quality reads. MACS2 (v2.2.6) was used for peak calling, with IgG as control. Differential peak analysis was performed using DiffBind (R v3.8.4) and DESeq2 (R v1.38.3). Peaks were annotated using ChIPseeker (R v1.43.0), and KEGG pathway enrichment analysis was conducted with ClusterProfiler (R v4.6.2). The scripts for CUT&Tag analysis have been deposited on GitHub and archived in Zenodo under DOI: 10.5281/zenodo.18043934, and Figshare (https://doi.org/10.6084/m9.figshare.30403936).
